# Design and Motion Analysis of a Soft-Limb Robot Inspired by Bacterial Flagella

**DOI:** 10.3390/biomimetics8030271

**Published:** 2023-06-26

**Authors:** Changlong Ye, Zhanpeng Liu, Suyang Yu, Zifu Fan, Yinchao Wang

**Affiliations:** 1 School of Mechatronics Engineering, Shenyang Aerospace University, Shenyang 110136, China; changlye@163.com (C.Y.); vulcanliu@outlook.com (Z.L.); wangyinchao2008@126.com (Y.W.); 2 School of Mechanical and Aerospace Engineering, Nanyang Technological University, Singapore 569830, Singapore; fanz0006@e.ntu.edu.sg

**Keywords:** tendon drive, leg–wheel mechanism, bioinspired robot, quadruped robot, gait planning

## Abstract

Soft robots demonstrate an impressive ability to adapt to objects and environments. However, current soft mobile robots often use a single mode of movement. This gives soft robots good locomotion performance in specific environments but poor performance in others. In this paper, we propose a leg–wheel mechanism inspired by bacterial flagella and use it to design a leg–wheel robot. This mechanism employs a tendon-driven continuum structure to replicate the bacterial flagellar filaments, while servo and gear components mimic the action of bacterial flagellar motors. By utilizing twisting and swinging motions of the continuum structure, the robot achieves both wheeled and legged locomotion. The paper provides comprehensive descriptions and detailed kinematic analysis of the mechanism and the robot. To verify the feasibility of the robot, a prototype was implemented, and experiments were performed on legged mode, wheeled mode, and post-overturning motion. The experimental results demonstrate that the robot can achieve legged and wheeled motions. Moreover, it is also demonstrated that the robot still has mobility after overturning. This expands the applicability scenarios of the current soft mobile robot.

## 1. Introduction

From the sea to the land, mollusks can be found everywhere, with examples in the ocean including jellyfish, octopi, and, squids. On land, examples include slugs, earthworms, and soft insect larvae. Their living environment shows a rich diversity: forests, mountains, rock piles, grasses, swamps, and even the sea [1]. Through their soft bodies and complex locomotor mechanisms, these organisms show strong environmental adaptability, as they moves freely in these living environments, climbing trees, swimming, burrowing, and bypassing obstacles [2]. Although a soft-bodied organism’s carapace appears to be just a thin rope, it possesses powerful functions: it can bear weight like a leg when moving forward, grab things like an arm when climbing, and be as flexible as a finger when grasping objects. Despite this structure’s seeming simplicity, powerful features are behind it [2].

Inspired by these soft organisms, humans have summarized the natural principles they contain, mimicked these natural organisms, and applied these principles to develop various bionic soft robots. Examples include bionic frog robots [3], bionic fish robots [4], bionic octopus robots [5], and bionic elephant trunk robots [6]. The soft robots are constructed using flexible materials and thus are mechanically flexible. This flexibility can bring many advantages to soft robots. For example, when a soft robot interacts with the external environment, it can be passively deformed, allowing for a larger contact area between the robot and the environment and better environmental adaptability [7]. This flexibility can even allow the robot to squeeze into tight gaps that are unreachable by traditional rigid robots [8,9]. The better environmental adaptability of soft robots expands the ability of humans to explore dangerous structures, extreme environments, and hard-to-reach spaces [10]. 

Soft robots working on land use a single mode of locomotion, consisting mainly of crawling, legged locomotion, and jumping [11]. The single mode of locomotion makes soft robots have good locomotion in specific environments but poor performance in other environments [12]. The application scenarios of soft robots are limited. Crawling and legged locomotion allow for flexibly avoiding obstacles in unstructured environments and adapting to complex terrain [12]. However, the slower gait and shorter step length in flatter environments lead to a large motion efficiency gap between soft and traditional wheeled robots. Jumping motion can quickly cover longer distances in a short time, enabling a robot to quickly pass obstacles or hazardous areas [11,12]. However, it may be less applicable in some scenarios that require precise movement or stable motion over a long duration [13]. Therefore, it is of great research significance to try to combine multiple motion modes to design a soft robot with multiple motion modes.

**Figure 1:**
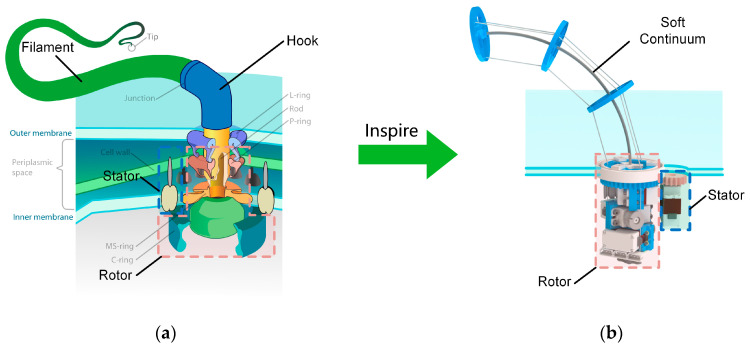
(**a**) Bacterial flagellum base diagram [18]. (**b**) The leg–wheel mechanism is inspired by bacterial flagella.

Leg–wheel robots are mobile robots with multiple forms of motion. Such robots combine the high energy efficiency of wheeled robots in flat terrain and the high mobility of legged robots in complex terrain [14]. However, few robots can achieve leg–wheel motion among the current soft robots. This may be because few biological structures in nature can continuously turn around [15], which makes it difficult for humans to learn how to design soft-body structures that can continuously turn around. The bacterial flagellum is one of the few biological structures in nature that can achieve continuous rotation [16]. The rotational motion of the bacterial flagellar filaments, driven by a motor at their base, propels the bacterium [17], as shown in Figure 1a.

This work introduces a novel leg–wheel mechanism called the BFWL module, inspired by bacterial flagella. The BFWL module incorporates a tendon-driven continuum structure as its soft-body component, resembling the flagellar filaments found in bacteria, as shown in Figure 1b. The robot achieves wheeled motion by inducing twisting movements in its soft parts, akin to the rotational motion of a bacterial flagellar filament driven by a base motor. Additionally, legged motion is accomplished by swinging the soft-body component. The subsequent sections of this paper are organized as follows: Section 2 details the mechanical design of the BFWL module and the BFWLbot. Section 3 presents the kinematic analysis. Section 4 presents simulation experiments performed on the BFWLbot. Section 5 shows the prototype of the BFWL module with BFWLbot and experiments performed on the prototype. Section 6 provides a conclusion and discusses future work.

## 2. Materials and Methods

### 2.1. Biological Characteristics

The bacterial flagellum is a helical filamentous supramolecular nanomachine that attaches to the bacterial body surface and extends outside the body [19]. The bacterial flagellum mainly consists of three major parts: a membrane-embedded basal body, a hook, and a filament [19,20]. The basal body is a complex assembly that functions as a motor, so it can be called the flagellar motor. The flagellar filament can reach tens of nanometers in length [21] and is functionally like a propeller [19,22]. The hook device is a transition structure connecting the flagellar filaments and the flagellar motor that is functionally like a universal joint and can achieve a large twist of the flagellar filaments through flexible conformational changes [19].

The flagellar motor is the power unit of the entire flagellar system. Although its molecular weight is only 1% of the total flagellar system, the flagellar motor is the engine for torque generation, transmission, and control of the bacterium’s orientation [22]. The flagellar motor consists of a rotor spanning the inner and outer membrane and a stator unit surrounding it. The stator unit converts chemical energy into mechanical energy and generates torque for the rotor unit. The rotor unit is connected to a hook unit, which causes the hook unit and the flagellar filaments to rotate. The flagellum’s rotation drives the bacterium’s movement [22,23].

### 2.2. Design of Leg–Wheel Mechanism

Inspired by the biological structure of bacterial flagella, the structure scheme of the BFWL module was designed. As shown in Figure 1b, the BFWL module uses a tendon-driven continuum structure to mimic the flagellar filament and hook device. The base of the continuum is designed with a pair of gears driven by a twisting motor that mimics the flagellar motor embedded in the cell membrane of bacteria. The torque generated by the twisting motor is transmitted to the tendon-driven continuum structure, which mimics the flagellar filament, to twist it and thus drive the BFWL module to produce wheeled motion. Three tendons on the continuum structure are controlled to keep the bending angle and rotation angle of the continuum constant during twisting.

**Figure 2:**
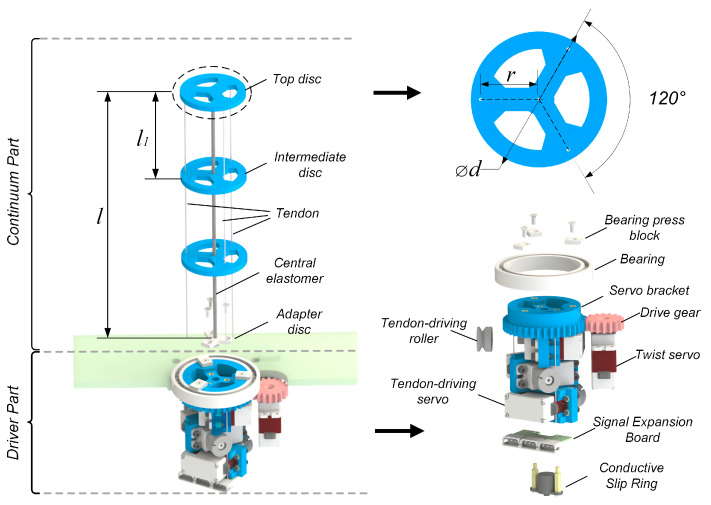
The detailed structure of the BFWL module.

The BFWL module comprises two parts: the continuum part and the driver part, as shown in Figure 2, which serve different functions. The continuum part interacts with the environment, while the driver part provides rotational power to the continuum part. The continuum part uses a glass fiber rod with a diameter of 1.8 mm and a length of 150 mm as the central elastomer. The continuum part comprises a top disc with a thickness of 3 mm, 2 intermediate discs with a thickness of 3 mm, an adapter disc, and 3 tendons made of ∅0.25 mm nylon rope. The top and intermediate discs are evenly spaced and arranged on the central elastomer, with both having 3 through-holes of 0.3 mm in diameter. The spacing between the discs is 47 mm. The 3 through-holes are distributed at 120° angular intervals on a circle with a diameter of 27 mm. The adapter disc connects the central elastomer to the servo bracket of the driver part.

The driver part employs 4 Feetch STS3032 servos as drive motors, each capable of producing a torque of up to 4.5 kg∙cm. In total, 3 of the servos are mounted on the servo bracket at 120° intervals and serve as tendon-driving motors. The servo bracket rotates with the continuum twisting, while the output shaft of each tendon-driving motor is equipped with a winding reel to drive the tendon, which has a diameter of 8 mm. The fourth servo, referred to as the twisting servo, is mounted on the robot frame and powers the wheel motion of the leg–wheel mechanism responsible for the twisting motion of the continuum. The output shaft of the twisting servo is fitted with a gear of module 1 and a tooth number of 18. The servo bracket, on the other hand, features a gear of module 1 and a tooth number of 38. The two gears engage to produce a reduction ratio of K = 38:18. The servo signal expansion board at the end of the servo bracket expands the single-servo signal into a 3-way signal. A conductive slip ring connects the single-servo signal line to the control board and prevents signal wire tangling, which can impede movement during continuous rotation of the leg–wheel mechanism.

**Figure 3:**
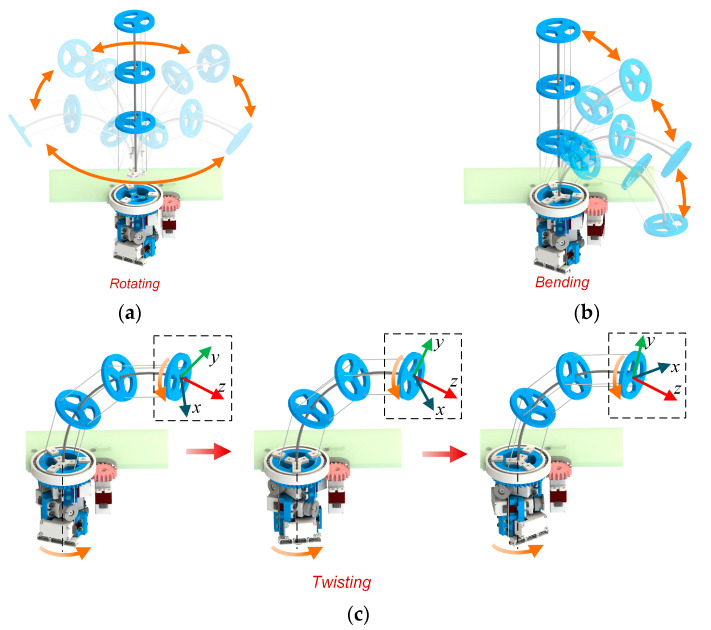
The BFWL module can perform three kinds of motion: (**a**) rotating; (**b**) bending; (**c**) twisting.

The continuum part will twist to perform wheeled motion, so the stiffness of the continuum part should be as uniform as possible. This makes the arrangement of the tendons in the continuum section need to be evenly arranged. To keep the tendon drive continuum in a fully driven state and to meet the need for even arrangement of tendons, the minimum number of tendons in the continuum part is three. The tendon routing of the tendon-driven continuum can be divided into parallel tendons and helical tendons [24]. When a parallel tendon is added so that the BFWL module has four parallel tendons, the degrees of freedom of the tendon-driven continuum do not increase [25,26]. The tendon-driving motor controls the length of the nylon rope, while the twisting motor controls the angle of the servo bracket. These motors operate based on the motor angle determined by inverse kinematics, enabling the control of the bending angle, bending direction, and twisting angle of the central elastomer, as shown in Figure 3.

### 2.3. Design of the Leg–Wheel Soft Robot

**Figure 4:**
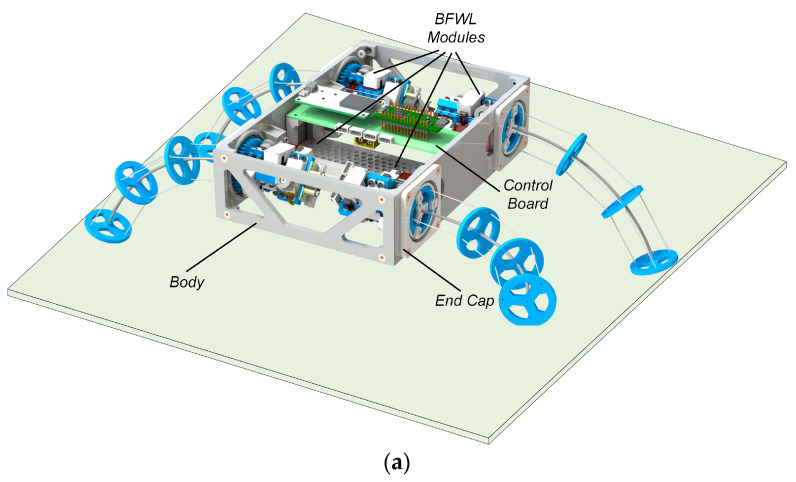

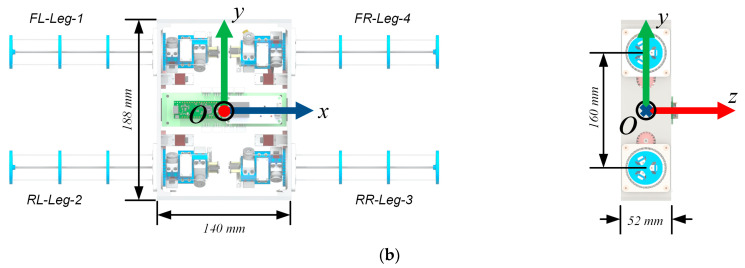
(**a**) The structural layout of BFWLbot. (**b**) The detailed size parameters of BFWLbot.

The leg–wheel soft robot, referred to as BFWLbot in this paper, is designed based on the BFWL module, as depicted in Figure 4a. The structure layout and detailed dimensions of the robot are presented in Figure 4b. The BFWLbot follows a quadrupedal configuration, with four sets of symmetrically distributed BFWL modules on each side. The main body of the BFWLbot is assembled using several sheet-like components, with the control board positioned at the center of the main frame. The BFWL modules are mounted within circular slots on the side sheets, where the inner wall of the slot accommodates the outer ring of the nylon bearing of the BFWL module. A rectangular end cap presses against the end face of the outer ring of the nylon bearing, allowing the BFWL module to be held in place. The specific parameters of the robot are summarized in Table 1.

The continuum part of the BFWL module can perform bending, rotating, and twisting motions. While the bending and rotation angles remain constant, controlling the increase or decrease in the twisting angle can drive the BFWL module to move. Multiple BFWL modules twist together to make the robot achieve wheel motion like that of a 4WD car. When the twisting angle is kept constant, regular control of the bending angle and rotation angle can make the end of the BFWL module swing. Multiple BFWL modules swinging together allow the robot to move like a quadruped with legs.

Different from the deformable leg–wheel mechanism of Wheel Transformer [27] and Turboquad [28,29], the BFWL module does not require a mode-switching process between wheel mode and leg mode, so a switching space is not required. This characteristic makes BFWLbot show better dexterity. The BFWLbot’s four legs are symmetrically distributed with respect to the center level of the robot’s body, so they can still work when the robot is overturned. This characteristic allows the BFWLbot to show better environmental adaptability.

## 3. Kinematic Analysis

### 3.1. Kinematic Modeling of Leg–Wheel Mechanism

**Figure 5:**
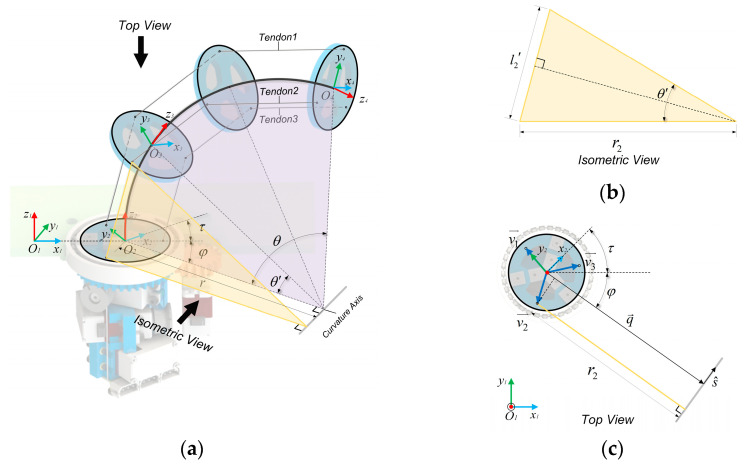
(**a**) The schematic diagram for kinematic modeling of the BFWL module. (**b**) Isomeric view of the diagram. (**c**) Top view of the diagram.

There are four frames defined on the BFWL module: the base frame${\{O}_{1}\}$, the servo bracket frame${\{O}_{2}\}$, the middle disc frame${\{O}_{3}\}$, and the end disc frame${\{O}_{4}\}$, as shown in Figure 5a.

The vector ${[\theta }_{1},\theta_{2},\theta_{3},\theta_{4}]$ comprises the rotational angles of the three tendon-driving motors and one twisting motor and serves as the actuator space parameter. Different from a traditional articulated robot, a continuum robot has no obvious joints, but the bending angle θ and rotation angle φ of the continuum are used to describe the robot configuration [25]. With the addition of twisting motion via the BFWL module, the twisting angle τ represents the twisting motion. The vector $\left[\theta ,\varphi ,\tau \right]$, consisting of these three angles, is the configuration space parameter. The spatial vector of the end disk $\left[R_{14},P_{14}\right]$ is the task space parameter [25]. 

The mapping $f_{2}$, which maps the configuration space to the pose space of the top disk of the robot, can be obtained through the following procedure. The length of the central elastomer $l_{c}$ is a fixed, and the central angle is θ. Therefore, the radius of curvature r can be obtained using Equation (1).

(1) $$r=\frac{l_{c}}{\theta }$$

The screw axis S can be obtained using Equations (2)–(4) [30].

(2) $$S=\left[\begin{array}{c} \hat{s}\\ -\hat{s}\times \vec{q} \end{array}\right]$$

(3) $$\vec{q}=Rot\left(\hat{z_{1}},\varphi \right)\left[\begin{array}{c} r\\ 0\\ 0 \end{array}\right]$$

(4) $$\hat{s}=Rot\left(\hat{z_{1}},\varphi \right)\left[\begin{array}{c} 0\\ 1\\ 0 \end{array}\right]$$

The expressions T12, T13, T14 of ${\{O}_{2}\}$,${\{O}_{3}\}$,${\{O}_{4}\}$ in ${\{O}_{1}\}$ can be obtained by Equation (5) [30].

(5) T12=Rotz1^,τ001T11T13=eSθ′T12T14=eSθT12

The mapping ${f_{1}}^{-1}$, which maps from configuration space to actuator space, can be obtained through the following procedure. In Figure 5c, $\vec{v_{1}}$, $\vec{v_{2}}$, and $\vec{v_{3}}$ are three vectors from the center of the disk pointing to the center of the through-hole and can be obtained using Equation (6).

(6) $$\left\{\begin{array}{c} \vec{v_{1}}=Rot\left(\hat{z_{1}},\tau \right)\left[\begin{array}{c} 0\\ r_{h}\\ 0 \end{array}\right]\\ \vec{v_{2}}=Rot\left(\hat{z_{1}},\tau +\frac{2\pi }{3}\right)\left[\begin{array}{c} 0\\ r_{h}\\ 0 \end{array}\right]\\ \vec{v_{3}}=Rot\left(\hat{z_{1}},\tau +\frac{4\pi }{3}\right)\left[\begin{array}{c} 0\\ r_{h}\\ 0 \end{array}\right] \end{array}\right.$$

The length of the i-th tendon $l_{i}$ can be determined using Equations (7) and (8), where $r_{i}$ denotes the length of the vertical line segment drawn from the center of the i-th through-hole to the bending axis of the continuum (i=1,2,3).

(7) $$r_{i}=r-\frac{\vec{v_{i}}\cdot \vec{q}}{\left\|\vec{q}\right\|}$$

(8) $$l_{i}=3{l_{i}}^{\prime }=6r_{i}sin\left(\frac{\theta^{\prime }}{2}\right)$$

In the initial state, the servo is positioned at zero, and the length of the tendon matches the length of central elastomer $l_{c}$. Consequently, the relationship between the angle of the tendon-driving motors $\theta_{i}$ and the length of the tendon $l_{i}$ is expressed as follows:

(9) $$\theta_{i}=\frac{\left(l_{i}-l_{c}\right)}{r_{\mathrm{reel}}}\left(i=1,\;2,\;3\right)$$

The relationship between the angle of the twisting motor $\theta_{4}$ and the twisting angle τ is as follows:

(10) $$\theta_{4}=\tau \times k$$

### 3.2. Contact Point Analysis

**Figure 6:**
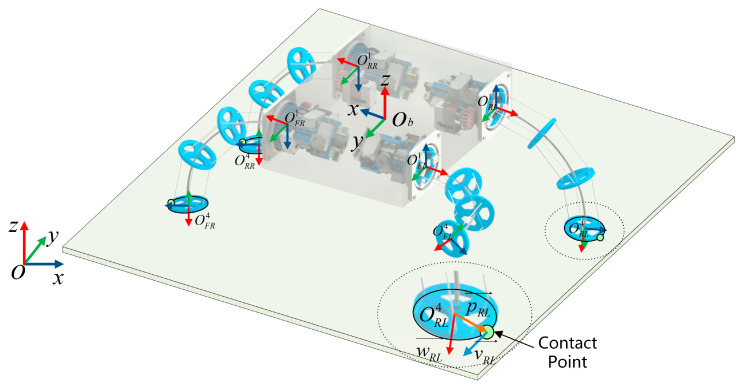
The definition of the frames on the robot and the contact point produced by the top disc of the rear-left leg.

The definition of the frames on the robot is shown in Figure 6. The body frame of the robot is $\left\{O_{b}\right\}$, and the origin of the frame is located at the geometric center of the robot. The base frame on i-th leg is denoted by $\left\{O_{i}^{1}\right\}$, where $i\epsilon \left\{FL,RL,RR,FR\right\}$, which denote left front, left rear, right rear, and right front, respectively [29]. Equations (11) and (12) can be used to obtain Tbi1. The configuration space parameters of i-th leg are $\left[\theta_{i},\varphi_{i},\tau_{i}\right]$, and Ti1i4 can be obtained using Equation (5). The frame of the top disc is $\left\{O_{i}^{4}\right\}$, which can be obtained using Equation (13). The coefficients in equations are summarized in Table 2.

(11) Tbi1=Rotyb^,αi·π2·Rotzb^,βi·π6pi101

(12) $$p_{i1}=\left[\begin{array}{c} \zeta_{i1}\cdot \frac{l_{\mathrm{width}}}{2}\\ \zeta_{i2}\cdot \frac{l_{\mathrm{length}}}{2}\\ 0 \end{array}\right]$$

(13) T=bi4T·i1i4Tbi1

The legs of a quadruped robot have two working states. The operating state in which the legs are in contact with the ground and propel the body forward is called the “Stance phase”. The state in which legs do not make contact with the ground but swing in the air to adjust their posture is called the “Swing phase” [31].

When a leg is in the stance phase, the top disc is in contact with the ground, producing a contact point, as shown in Figure 6. This point can be approximated as the lowest point of the outermost circle of the top disc in the $\hat{z}$ direction in the robot’s body frame $\left\{O_{b}\right\}.$ The lowest point of a circle in a space can be derived by the following method.

**Figure 7:**
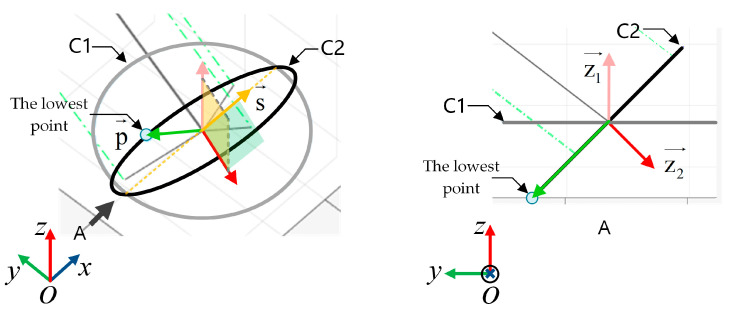
The schematic diagram for solving the lowest point on a circle.

The lowest point on a circle is shown in Figure 7. C1 is a circle in the x-y plane. The normal vector to the x-y plane is $\hat{z_{1}}$. C2 is a circle in some known plane in three-dimensional space. The normal vector of this plane is $\hat{z_{2}}$. The centers of these two circles are the same point. Thus, C2 can be regarded as a circle obtained by rotating C1 around an axis of rotation $\vec{s}$. The vector of the rotation axis $\vec{s}$ can be obtained by Equation (14).

(14) $$\vec{s}=\hat{z_{1}}\;\times \hat{z_{2}}$$

The lowest and highest points on circle C2 are the two points farthest from the rotation axis. The vector with the center of the circle pointing to the lowest point $\vec{p}$ and the vector with the center of the circle pointing to the highest point $\vec{o}$ can be obtained by Equations (15) and (16), respectively.

(15) $$\vec{p}=\frac{\vec{s}\times \hat{z_{2}}}{\left\|\vec{s}\times \hat{z_{2}}\right\|}\;\cdot \frac{d}{2}$$

(16) $$\vec{o}=\frac{\hat{z_{2}}\times \;\vec{s}}{\left\|\hat{z_{2}}\times \;\vec{s}\right\|}\;\cdot \frac{d}{2}$$

Combined with the structure of the BFWLbot, the expression of the vector $\vec{p}$ on the i-th leg in the robot body frame $\left\{O_{b}\right\}$ can be obtained by using Equation (17). $\hat{z_{i4}}$ is the z-axis unit vector of the top disc on the i-th leg, and $\vec{w_{i}}$ is the angular velocity vector of the top disc on that leg. Equation (21) solves for the linear velocity $\vec{v_{i}}$ of the top disc at that point.

(17) $$\vec{p_{i}}=\frac{\vec{s}\times \hat{z_{2}}}{\left\|\vec{s}\times \hat{z_{2}}\right\|}\;\cdot \frac{d}{2}=\frac{\hat{z_{b}}\times \hat{z_{i4}}\times \hat{z_{i4}}}{\left\|\hat{z_{b}}\times \hat{z_{i4}}\times \hat{z_{i4}}\right\|}\;\cdot \frac{d}{2}$$

(18) $$\vec{v_{i}}=\vec{w_{i}}\times \vec{p_{i}}$$

### 3.3. Kinematic Modeling of Wheeled Motion

**Figure 8:**
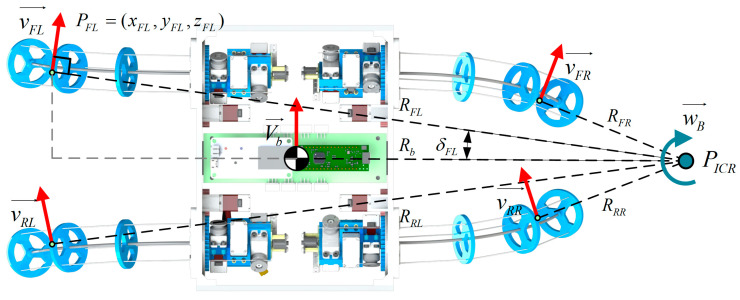
Steering of the BFWLbot when it is operated in wheeled mode.

The BFWLbot can realize various wheel motions by using different values of curve parameters. In this section, one of the turning modes, as shown in Figure 8, is chosen to analyze the wheel motion of BFWLbot. The velocity instant center of the robot $P_{\mathrm{ICR}}$ in this mode is located on the $\hat{x}$ axis of the robot body frame. The kinematic parameters of the robot on a flat surface can be expressed in terms of $V_{b}$, which can be calculated using Equation (19) [30]. The instantaneous center of velocity $P_{\mathrm{ICR}}$ and the radius of rotation $R_{B}$ of the robot can be calculated using Equation (20).

(19) $$V_{b}=\left[\begin{array}{c} \vec{w_{b}}\\ \vec{v_{b}} \end{array}\right]$$

(20) $$R_{b}=\frac{\left\|\vec{v_{b}}\right\|}{\left\|\vec{w_{b}}\right\|}$$

$P_{i}=[x_{i},y_{i},z_{i}]$ is the expression of the contact point of the i−th leg in $\left\{O_{b}\right\}$. The vertical segment $R_{i}^{}$ of $\vec{v_{i}}$ is the radius of rotation at the contact point $P_{i}$, and its endpoints are the contact point $P_{i}$ and the instantaneous center $P_{ICR}$. The angle between $R_{i}^{}$ and $R_{b}$ is $\delta_{i}$. When $P_{i}$ and $\vec{v_{i}}$ are known, Equations (21) and (22) can be used to determine $\delta_{i}$ and $R_{i}^{}$, respectively.

(21) $$\delta_{i}=\mathrm{acos}\left(\frac{\hat{y_{b}}\cdot \vec{v_{i}}}{\left\|\vec{v_{i}}\right\|}\right)$$

(22) $$R_{i}^{}=\frac{\left|y_{i}\right|}{\sin\left(\delta_{i}\right)}$$

The radius of the robot’s rotation $R_{b}$ is related to $R_{i}^{}$,$\delta_{i}$ as shown in Equation (23). Combining Equation (21) with Equation (22), it is known that $R_{i}^{}$,$\delta_{i}$ is determined by the continuum curve parameters $\theta_{i}$ and $\varphi_{i}$, so the function H can be used to represent $R_{b}$. After calculation, the function H contains many nonlinear parts, which will be very complicated when $\theta_{i}$ and $\varphi_{i}$ need to be calculated from a given $R_{b}$. Therefore, function fitting is used to simplify the function H to $H^{\prime }$.

(23) $$R_{b}=\frac{\left|y_{i}\right|}{\tan\left(\delta_{i}\right)}+x_{i}=H\left(\theta_{i},\varphi_{i}\right)\approx H^{\prime }\left(\theta_{i},\varphi_{i}\right)$$

When the target turning radius $R_{b}$ is obtained by using Equation (20), it can be brought into Equation (23) to obtain the implicit functions of $\theta_{i}$ and $\varphi_{i}$ that satisfy the conditions. Then, the appropriate parameters of $\theta_{i}$ and $\varphi_{i}$ are selected and brought into Equation (24), and the twisting speed $w_{i}$ of the i-th leg can be calculated.

(24) $$w_{i}=\frac{\left\|\vec{v_{i}}\right\|}{r_{\mathrm{disk}}^{}}=\frac{\left\|\vec{w_{b}}\right\|\times R_{i}^{}}{r_{\mathrm{disk}}^{}}=\dot{\tau }$$

### 3.4. Gait Planning

**Figure 9:**
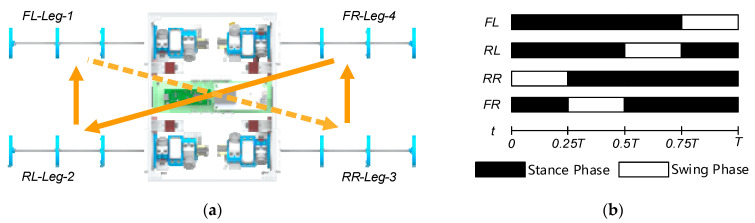
(**a**) Leg lift sequence diagram and (**b**) gait diagram of BFWLbot performing crawling gait.

The gait of a robot is determined by the end trajectory of a single foot and the phase relationship between the feet [32]. Quadrupeds are in a stable three-legged support state for each walk in a slower walking state, so their stability is ensured. This gait is called crawling gait. The crawling gait is a four-beat gait in which only one leg is lifted at a time, in the order of front-left, rear-right, front-right, and rear-left leg lifts, and the other three support legs form a triangular support area to make the robot achieve stable walking. This crawling gait is also used for the leg motion analysis in this paper. The leg-lifting sequence of BFWLbot can be expressed as RR-FR-RL-FL, as shown in Figure 9a.

**Figure 10:**
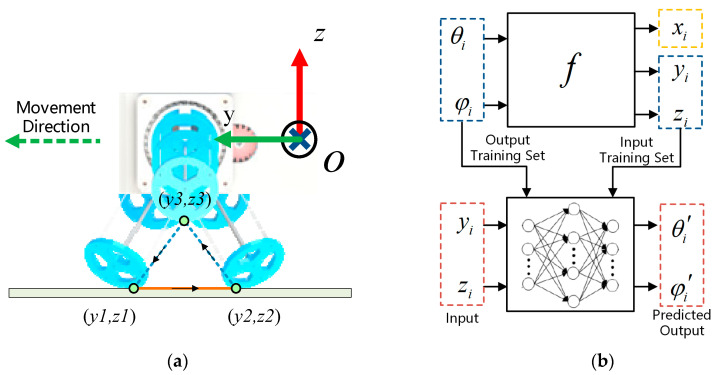
(**a**) The trajectory of the lowest point of the top disc when the BFWLbot is crawling. (**b**) BP neural network is used to fit the mapping from $\left[y_{i},z_{i}\right]$ to ${[\theta }_{i},\varphi_{i}]$.

The black square in Figure 9b indicates that the leg is in the stance phase. In this phase, the leg is in contact with the ground to support the torso and propel the torso forward. The trajectory of the contact point between the leg and the ground should remain horizontal, such as the trajectory from $\left(y_{1},z_{1}\right)$ to $\left(y_{2},z_{2}\right)$ in Figure 10a. The white square indicates that the leg is in the swing phase. When the leg is in this phase, it lifts and swings in the air. Its end trajectory should quickly swing from the end of the trajectory during the support phase to the start of the trajectory, such as the trajectory from $\left(y_{2},z_{2}\right)$ to $\left(y_{3},z_{3}\right)$ to $\left(y_{1},z_{1}\right)$ in Figure 10a [32].

**Figure 11:**
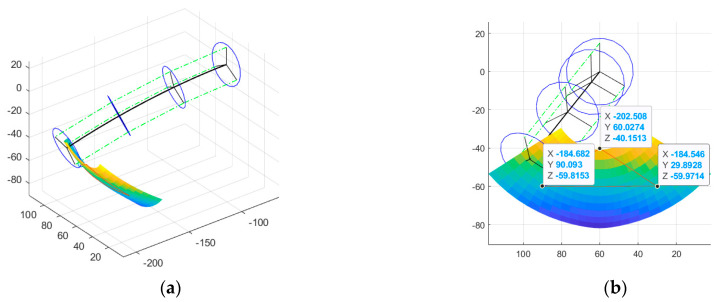
(**a**) The motion space of the lowest point when $\theta_{i}\in \left[-70,-20\right]$ and $\varphi_{i}\in \left[-60,60\right]$. (**b**) The red trajectory is the lowest point trajectory obtained after fitting $f^{-1}$ using BP neural network with ${{[\theta }_{i}}^{\prime },{\varphi_{i}}^{\prime }]$ as the continuum parameter.

The curve parameter $\theta_{i}$ is chosen from the range $\left[-70,-20\right]$, and $\varphi_{i}$ is chosen from the range $\left[-60,60\right]$. By combining these parameters with Equation (17), the lowest point of the leg $\left[x_{i},y_{i},z_{i}\right]$ under these two ranges is calculated and is plotted as the motion space diagram shown in Figure 11a. Suitable $\left(y_{1},z_{1}\right)$, $\left(y_{2},z_{2}\right)$, $\left(y_{3},z_{3}\right)$ are selected in the motion space to determine the whole trajectory. The values of $\left(y_{1},z_{1}\right)$, $\left(y_{2},z_{2}\right)$, and $\left(y_{3},z_{3}\right)$ are shown in Table 3.

$P_{i}=[x_{i},y_{i},z_{i}]$ is determined by $\theta_{i}$ and $\varphi_{i}$, and this relationship can be expressed by the function f, as shown in Figure 10b. Since f also contains many nonlinear parts, it is not easy to find the inverse solution directly. Therefore, BP neural network is chosen to fit the mapping from $\left[y_{i},z_{i}\right]$ to ${[\theta }_{i},\varphi_{i}]$, which is used to realize the computation of ${{[\theta }_{i}}^{\prime },{\varphi_{i}}^{\prime }]$ sequence from the given $\left[y_{i},z_{i}\right]$ sequence. Then, the ${{[\theta }_{i}}^{\prime },{\varphi_{i}}^{\prime }]$ sequence is used as input of BFWL module continuum parameter to control the lowest point of the BFWL module to draw the red trajectory, as shown in Figure 11b.

## 4. Verification by Simulation

### 4.1. Wheeled Motion Simulation

**Figure 12:**
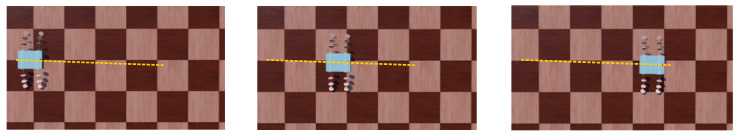
Snapshots of BFWLbot performing straightforward wheeled motion in the simulation.

The robot’s straightforward wheeled motion is first simulated. In the simulation, the bending angle of all 4 BFWL modules is chosen to be −50°, the rotation angle is chosen to be 0°, and the twisting speed $\dot{\tau_{i}}$ is 5°/s. The robot is simulated with these parameters, and snapshots of the simulation video are shown in Figure 12. The robot demonstrates good motion in the straightforward wheeled motion mode.

The robot’s wheeled steering motion is then simulated by integrating the kinematic model in Section 3.3. The target motion linear velocity of the robot is chosen as 10mm/s, and the target turning radius of the robot is chosen as 300 mm. The implicit function relationship between $\theta_{i}$ and $\varphi_{i}$ for each leg is obtained by substituting them into Equation (23). The rotation angle $\varphi_{i}$ of each leg is solved by making $\theta_{i}=-50$ and is substituted into Equation (24) to obtain the twisting velocity of each BFWL module, as shown in Table 4.

**Figure 13:**
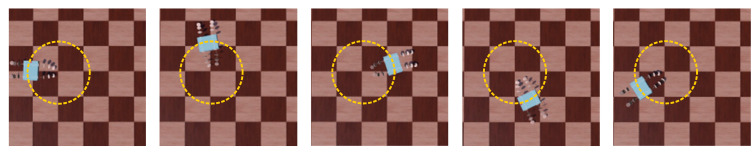
Snapshots of BFWLbot performing wheeled steering motion in the simulation.

**Figure 14:**
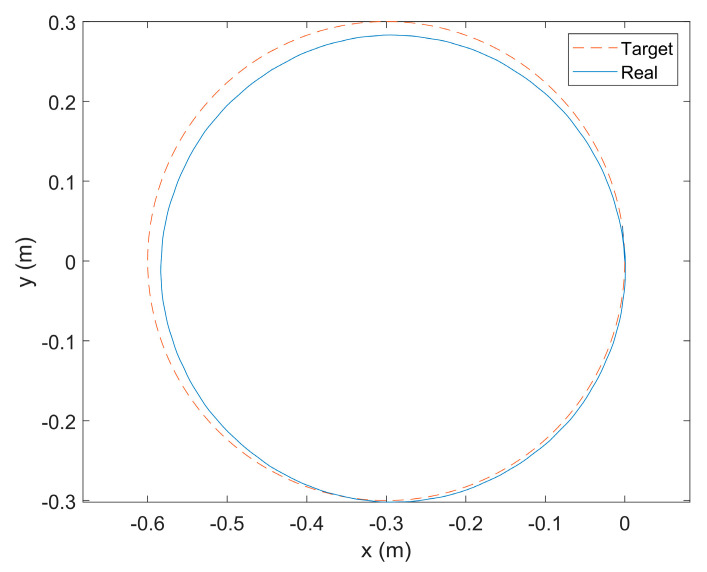
The red trajectory is the target trajectory, and the blue trajectory is the actual trajectory of the BFWLbot performing wheeled steering.

Simulation video snapshots are shown in Figure 13. The trajectory of the robot is recorded as shown in Figure 14. The red trajectory in the figure is the target trajectory, and the blue trajectory is the actual trajectory of the robot. The same motion trend can be seen, showing the blue trajectory can fit the red trajectory well. The simulation results verify the correctness of the wheeled kinematic model in Section 3.3.

### 4.2. Gait Simulation

**Figure 15:**
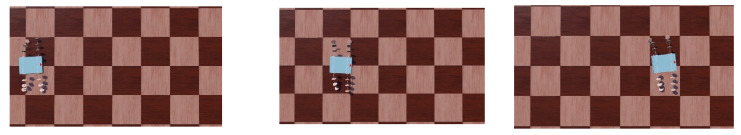
Snapshots of BFWLbot performing crawling gait in the simulation.

The ${[\theta }_{i},\varphi_{i}]$ sequence obtained from the previous gait planning is used as the control parameter input for each leg. The simulation of the robot is executed, and snapshots of the simulation video are shown in Figure 15. The simulation results show that the BFWLbot achieves forward walking motion with the gait derived in Section 3.4.

**Figure 16:**
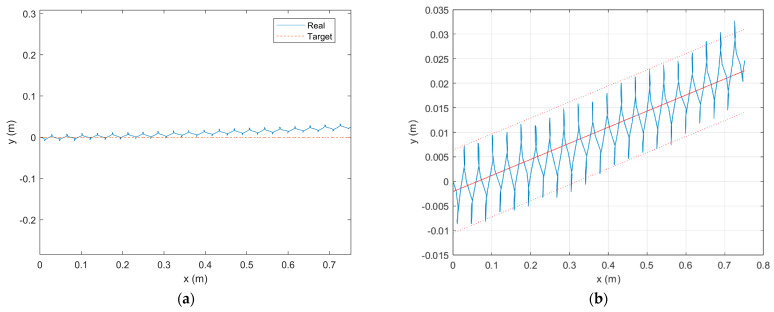
(**a**) The red trajectory is the target trajectory, and the blue trajectory is the actual trajectory of the BFWLbot performing a crawling gait. (**b**) The trajectory is fitted by a polynomial function and exhibits first-order linearity.

The trajectories of the robot were recorded and shown in Figure 16a. The red trajectory in the figure is the target trajectory of the robot, and the blue trajectory is the trajectory generated by the robot performing legged motion. Due to the structural characteristics of the BFWLbot, the blue trajectory exhibits regular fluctuations in the y-direction, while it shows a walking forward motion trend in the x-direction.

As the robot moves forward in the x-direction, a cumulative motion error appears in the y-direction. A polynomial fit of the trajectory was performed to obtain Figure 16b, and it was found that the trajectory exhibited first-order linearity. It is speculated that the error arises from the robot’s overall forward direction due to its posture at the starting position. The simulation results verify the correctness of the motion law obtained from the gait planning in Section 3.4.

## 5. Experimental Tests

### 5.1. Wheeled Motion Experiment

**Figure 17:**
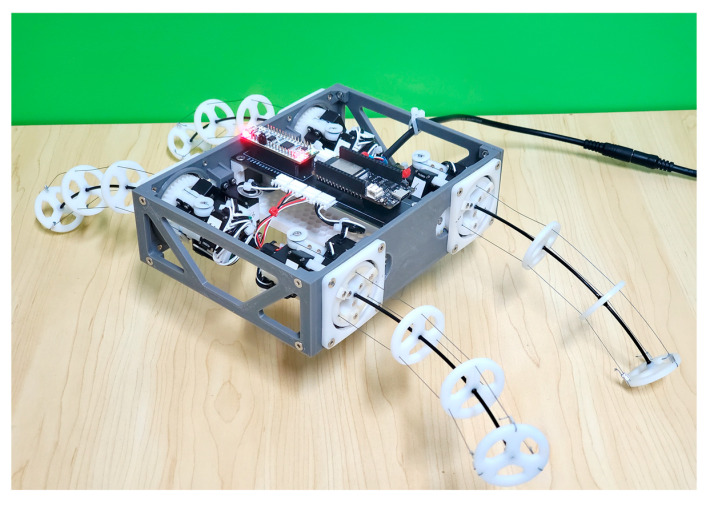
The prototype of the BFWLbot.

Based on the layout of the structure in Figure 4a, the prototype of the BFWLbot is built as shown in Figure 17. In the experiments on the robot prototype, Aruco tags and OpenCV are used for robot trajectory tracking. An Aruco tag with ID 1 was attached to the front of the top of the robot, and an Aruco tag with ID 2 was attached to the back of its top. The Aruco tags with ID 3 and 4 were attached to the left and right of the BFWLbot prototype body, respectively. These labels are in 6 × 6 format and have a size of 30 × 30 mm.

**Figure 18:**
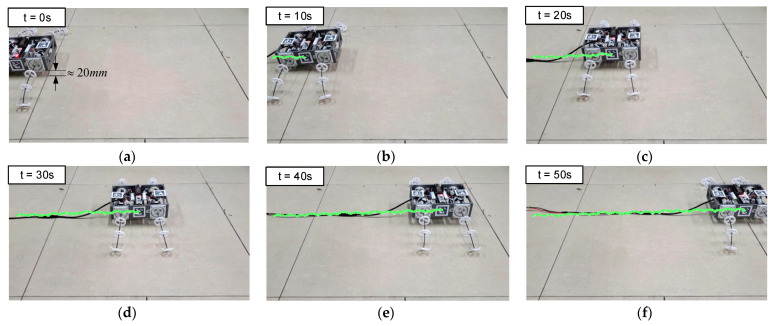
Snapshots of BFWLbot performing straightforward wheeled motion in the experiment. (**a**) t = 0 s; (**b**) t = 10 s; (**c**) t = 20 s; (**d**) t = 30 s; (**e**) t = 40 s; (**f**) t = 50 s.

To verify its feasibility for wheeled motion, the first experiment was on the straight wheeled motion of the BFWLbot. The parameters in Table 5 were used as the input of motion parameters for its four BFWL modules, and the robot’s body was lifted about 20 mm from the ground at t = 0 s. Four BFWL modules were twisting to propel the robot’s motion. The experimental process was captured on camera, and the Aruco tag with ID 3 on the BFWLbot was detected in real time using OpenCV, followed by plotting the robot motion trajectory on a graph using Python. The motion process is summarized in Figure 18.

Based on the trajectory in the figure, the BFWLbot prototype successfully completes the straight wheel motion, but its body appears to have regular upward and downward fluctuations. The reason for this phenomenon is that the stiffness anisotropy existing in the tendon-driven continuum structure makes the bending stiffness of the BFWL module variable in different directions. As a result, the height of the BFWL module changes periodically with the change in the twisting angle τ during the twisting process. In the future, higher-level motion control algorithms can be further developed to achieve suppression of this regular vibration to make the BFWLbot operate more smoothly.

**Figure 19:**
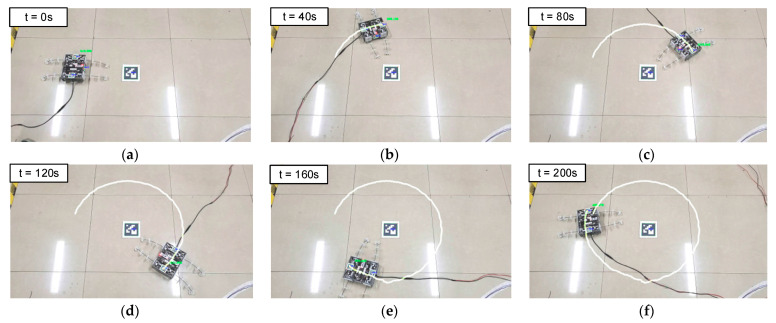
Snapshots of BFWLbot performing wheeled steering motion in the experiment. (**a**) t = 0 s; (**b**) t = 40 s; (**c**) t = 80 s; (**d**) t = 120 s; (**e**) t = 160 s; (**f**) t = 200 s.

The next experiment was on the wheeled steering motion of the BFWLbot. The parameters in Table 6 were used as input for the motion parameters of the four BFWL modules. Experiments were conducted using these parameters as input parameters for the motion of the BFWLbot. The experimental process was filmed using a camera. The Aruco tag with ID 1 on the BFWLbot was captured in real time using OpenCV, which was followed by plotting the robot motion trajectory on a graph using Python. The motion process is summarized in Figure 19.

The BFWLbot moves with a circular trajectory. Based on the estimation of the position of the Aruco tag with ID 1, the motion radius is about 385.8 mm, and there is a gap of 31.3 mm between the starting point and the ending point. There is some deviation from the expected circular trajectory. After further analysis, it is concluded that this error is mainly due to the characteristics of the tendon-driven continuum, such as the deformation of the continuum and the change in the bending angle, which can lead to errors during the robot’s motion. Meanwhile, the mechanical characteristics of the tendon-driven continuum also impact the accuracy of its motion trajectory. Therefore, the softness of the legs needs to be considered in practical applications, and the improvement of the motion accuracy and stability of the robot will be achieved by controlling and optimizing such characteristics accordingly.

### 5.2. Gait Experiment

**Figure 20:**
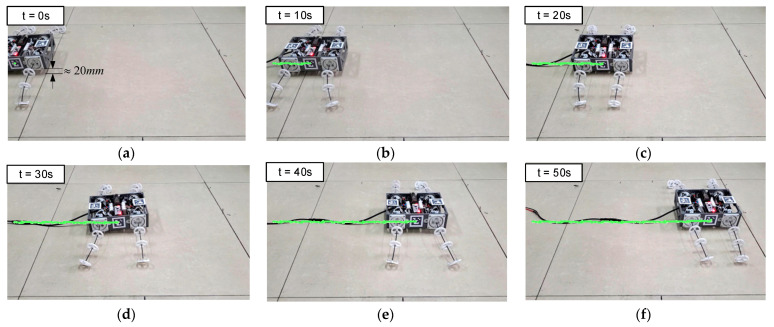
Snapshots of BFWLbot performing crawling gait in the experiment. (**a**) t = 0 s; (**b**) t = 10 s; (**c**) t = 20 s; (**d**) t = 30 s; (**e**) t = 40 s; (**f**) t = 50 s.

To validate the correctness of the gait planning, the BFWLbot executed its quadrupedal swinging motion using the derived gait pattern described in Section 3.4. At t = 0 s, the robot’s body was lifted approximately 20 mm above the ground level. The experiment was recorded using a video camera, and real-time detection of the Aruco tag with ID 3 on the BFWLbot was performed using OpenCV. The robot’s motion trajectory was then plotted using Python, as illustrated in Figure 20.

The trajectory in the figure shows that the BFWLbot prototype successfully achieved legged motion, and the Aruco tag on the robot traced a relatively straight trajectory. This shows that the robot can complete legged motion and confirms the effectiveness of the prototype. Through this experiment, the feasibility of BFWLbot’s legged motion at the prototype level has been verified and basic data has been provided for subsequent experiments. This means that the design and implementation of BFWLbot will have broader application prospects and can be used in various scenarios that require legged motion, such as walking or crawling in uneven or complex terrain.

### 5.3. Post-Overturning Motion Experiment

Robot overturning is a common cause of robots not working normally during robot motion, especially in complex environments, where robots are often faced with unpredictable obstacles and terrain that can cause them to overturn [24]. Robot overturning not only causes damage and stops the robot from working but also may lead to safety risks in dangerous environments, so it is important to resume work quickly after the robot is overturned.

**Figure 21:**
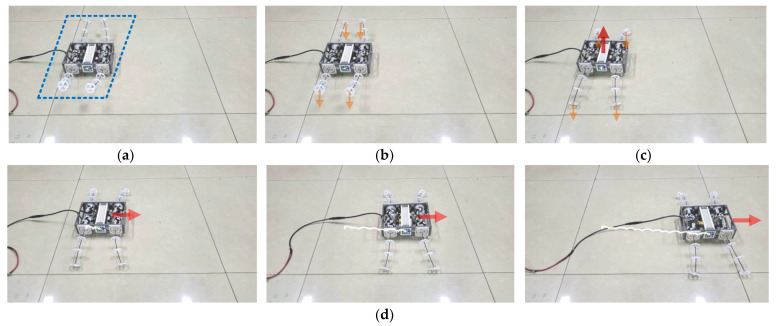
Snapshots of the motion of BFWLbot after overturning in the experiment. (**a**) BFWLbot was flipped to the bottom-side-up state. (**b**) The quadruped modules of the BFWLbot bend in the opposite direction. (**c**) The quadruped module gradually contacts the ground and supports the robot body off the ground. (**d**) BFWLbot continues to move normally.

BFWLbot is a structurally symmetric robot. Based on the analysis of the structural principle, when it overturns, BFWLbot can still interact with the environment by changing the legs’ rotation or bending angles. Therefore, this paper will simulate the BFWLbot in the case of overturning during operation and experiment with whether it can continue to move normally after the overturning. The experimental process is also filmed with a video camera, and the Aruco tag with ID 4 on the BFWLbot is detected in real time using OpenCV, after which the robot motion trajectory is drawn on a graph using Python. The motion process is summarized in Figure 21.

As shown in Figure 21, the BFWLbot was flipped to the bottom-side-up state on a flat surface, and the experiment started. The bending angle of the controlled quadruped module changed from −50° to 50°, so the quadruped module of the BFWLbot changed from the state shown in Figure 21a to the state shown in Figure 21c. The quadruped module gradually contacts the ground and supports the robot body off the ground. Finally, the wheeled motion program is started, as shown in Figure 21d, and the BFWLbot successfully performs wheeled straight motion.

Based on the above process, BFWLbot can change the working position of a leg by changing the rotation angle or bending angle of the leg after overturn and continue to move normally. This characteristic shows that the BFWLbot has good environmental adaptability and will not stop working due to overturning in the face of complex environments. This is very important for the robot in practical applications.

## 6. Discussion

Soft robots exhibit flexibility due to their bodies being constructed from deformable materials, enabling them to navigate confined spaces and traverse uneven terrain with ease. This paper introduces a novel leg–wheel mechanism inspired by bacterial flagella. We designed a soft-limb robot named the BFWLbot based on this mechanism, capable of executing both wheeled and legged motions.

In contrast to existing soft-limb robots [11], the BFWLbot eliminates the need for repetitive lifting of its four limbs while moving on flat terrain by incorporating wheeled motion. Consequently, it can move forward or change direction directly using wheeled motion, resulting in reduced energy consumption. With the integration of two locomotion modes, the BFWLbot possesses redundancy in its movements. In case of locomotion failure or limited space for a specific locomotion mode, the BFWLbot can rely on the alternative mode to maintain its movement. For instance, when the robot encounters a confined environment where leg swinging is limited due to insufficient working space, it can transition to the wheeled locomotion mode to maintain its mobility. This redundancy improves the reliability and robustness of the robot, making it less susceptible to a single point of failure.

The BFWLbot has flexibility in its limbs and symmetry in its overall structure. As compared to soft-limb robots whose legs can only function on one side of the body [33,34], the BFWLbot can control its legs to bend backward to reestablish contact with the ground after flipping. This feature enhances the robustness of the soft-limb robot, enabling it to navigate complex environments more effectively. Benefiting from the flexibility of its limbs, the BFWLbot can control the bending angle of each limb to change its body height off the ground. This capability enables the BFWLbot to more effectively navigate obstacles and gaps of varying heights in the environment than leg–wheel robots with fixed working heights [14].

Robots usually have better steadiness during wheeled motion than during legged motion. Observing the motion trajectory of the BFWLbot in wheeled motion and legged motion reveals that its steadiness in wheeled motion does not surpass that in legged motion. This phenomenon arises from the stiffness anisotropy of the tendon-driven continuum structure, leading to varying bending stiffness of the BFWL module in different directions. As a result, the robot's height from the ground exhibits regular undulations with the changing twisting angle τ. This undulation has a potential impact on the sensors that may be mounted on the robot in the future. In future versions, this undulation needs to be inhibited by improving the structure, applying a more accurate continuum-modeling approach [35], and implementing advanced motion control algorithms [36] simultaneously.

The BFWLbot benefits from the flexibility of the soft limbs, but it also faces limitations. The flexibility of the soft limbs results in a lower load capacity for the robot compared to rigid robots [37]. In our ongoing experiments, we found that the current prototype can carry a 361 g load. However, as we consider the possibility of future research involving an amphibious robot based on the BFWLbot design, where the discs function as propellers, the sealing of the robot will bring more additional self-weight. This requires us to increase the load capacity of the robot in future versions to achieve a balance between environmental adaptability and functionality of this robot. The current control method of the tendon in this work is position control. The flexibility of the robot under this control method comes entirely from the mechanical structure. The hybrid force-position control [38] of the continuum can add controlled flexibility to the limbs. This controlled flexibility can also compensate for the stiffness anisotropy of the tendon-driven continuum structure. Future research will explore new tendon control methods to further enhance the soft limbs' ability to interact with the environment safely.

## 7. Conclusions

This paper presents a leg–wheel mechanism inspired by bacterial flagella to enhance the mobility of soft-limb robots. The mechanism uses a tendon-driven continuum structure to mimic bacterial flagellar filaments and hooks and uses servos and gears to mimic bacterial flagellar motors. The mechanism achieves wheeled motion by twisting the continuum structure, while legged motion is performed by swinging the continuum structure. In contrast to the deformable leg–wheel structure, this mechanism is more flexible, as it eliminates the need for switching between the wheel and the leg. Based on this mechanism, a leg–wheel robot is designed. The robot consists of four leg–wheel mechanisms inspired by bacterial flagella. The symmetrical layout of the mechanisms with respect to the robot's centerline enables the robot to maintain normal movement even after overturning. To thoroughly analyze the robot’s motion, we conducted a kinematic analysis. The kinematics model of wheeled motion and gait of legged motion was obtained. After constructing a prototype of the robot, we conducted experiments to evaluate its performance. The experimental results show that the soft robot designed in this work has the capability to achieve legged and wheeled motion. Furthermore, the results demonstrate that the robot remains maneuverable even after overturning, thereby expanding the potential applications of soft robots.

## Figures and Tables

**Table 1:** The specific parameters of the robot.

Parameters	Values
Length (Body)	188 mm
Length (Hip-to-hip)	160 mm
Width (Body)	140 mm
Width (Tip-to-tip)	450 mm
Height	52 mm
Weight	715 g

**Table 2:** The coefficients in equations.

i	$\alpha_{i}$	$\beta_{i}$	$\zeta_{i1}$	$\zeta_{i2}$
FL	−1	1	−1	1
RL	−1	1	−1	−1
RR	1	−1	1	−1
FR	1	−1	1	1

**Table 3:** Position of points on the trajectory.

i	$y_{i}\;\left(\mathrm{mm}\right)$	$z_{i}\;\left(\mathrm{mm}\right)$
1	90	−60
2	30	−60
3	60	−40

**Table 4:** Parameters calculated from the kinematic model of the wheel motion to be used in the simulation.

i	$\theta_{i}\;\left({}^{\circ}\right)$	$\varphi_{i}\;\left({}^{\circ}\right)$	$R_{i}\;\left(\mathrm{mm}\right)$	$\dot{\tau_{i}}\;\left({}^{\circ}/s\right)$
*FL*	−50	−6.6286	475.8245	9.063
*RL*	−50	6.6286	475.8245	9.063
*RR*	−50	19.5868	96.9410	1.846
*FR*	−50	−19.5868	96.9410	1.846

**Table 5:** Parameters applied in the straightforward wheeled motion experiment.

i	$\theta_{i}\;\left({}^{\circ}\right)$	$\varphi_{i}\;\left({}^{\circ}\right)$	$\dot{\tau_{i}}\;\left({}^{\circ}/s\right)$
*FL*	−50	10	10
*RL*	−50	−10	10
*RR*	−50	−10	−10
*FR*	−50	10	−10

**Table 6:** Parameters applied in the wheeled steering motion experiment.

i	$\theta_{i}\;\left({}^{\circ}\right)$	$\varphi_{i}\;\left({}^{\circ}\right)$	$\dot{\tau_{i}}\;\left({}^{\circ}/s\right)$
*FL*	−50	−13.8865	49.4921
*RL*	−50	13.8865	49.4921
*RR*	−50	5.8975	−14.4786
*FR*	−50	−5.8975	−14.4786

## Data Availability

The data presented in this study are available on request from the corresponding author.
